# Supplementary material for: wgbstools: a computational suite for DNA methylation sequencing data analysis
Source: Life Sci Alliance. 2026 Jan 29;9(4):e202503514. doi: 10.26508/lsa.202503514 (PMC12861688; doi:10.26508/lsa.202503514)
Supplement: Supplementary file 1 [file LSA-2025-03514_TableS1.docx]

**Supplemental Table Legends**

**Table S1.** The datasets used in Figure 1 to compare format sizes.

**Supplemental Tables**

**Table S1.**

| **Dataset** | **Method** | **Num of samples** | **Mean cover.** | **BAM size** | **bigwig size** | **pat size** | **Compression factor** |
| --- | --- | --- | --- | --- | --- | --- | --- |
| Loyfer et al., 2023 | WGBS | n=205 | 30x | 100.7Gb | 239.9Mb | 302Mb | **342.1** |
| Roadmap [(Roadmap Epigenomics Consortium et al. 2015)](https://paperpile.com/c/lWSBAb/bOfT5) | WGBS | n=91 | 32x | 127.6Gb | 248.7Mb | 256.4Mb | **488.1** |
| METABRIC [(Batra et al. 2021)](https://paperpile.com/c/lWSBAb/odZx) | RRBS | n=1,782 | 2x | 1.7Gb | 44.7Mb | 12.8Mb | **137.2** |
| Cheng et al. [(Cheng et al. 2021)](https://paperpile.com/c/lWSBAb/wkdP) | WGBS | n=52 | 1.5x | 5.1Gb | 136.4Mb | 45.5Mb | **113.7** |
