# Supplementary material for: wgbstools: a computational suite for DNA methylation sequencing data analysis
Source: Life Sci Alliance. 2026 Jan 29;9(4):e202503514. doi: 10.26508/lsa.202503514 (PMC12861688; doi:10.26508/lsa.202503514)
Supplement: Supplementary file 2 [file LSA-2025-03514_TableS2.docx]

**Supplemental Table Legends**

**Table S2.** Comparison of available features in wgbstools and other software tools.

**Supplemental Tables**

**Table S2.**

|  | wgbstools | biscuit | DNMTools |
| --- | --- | --- | --- |
| Alignment |  | ✓ | ✓ |
| Supports any aligner | ✓ | ✓ |  |
| Compact read representation | ✓ | ✓ | ✓ |
| Compact representation of beta values | ✓ |  |  |
| Fast random access | ✓ | ✓ |  |
| Conversion to standard formats (e.g., bigwig) | ✓ | ✓ | ✓ |
| Pairwise similarity visualization | ✓ |  |  |
| Single-sample visualization | ✓ | ✓ |  |
| Visualization of multiple samples | ✓ |  |  |
| Merge, mix, subsampling of reads | ✓ | ✓ |  |
| DMR calling | ✓ |  | ✓ |
| de novo SNP calling |  | ✓ |  |
| Split bam files by methylation | ✓ |  |  |
| Split bam files by allele/SNP | ✓ |  |  |
| Identification of partially meth. domains (PMDs) |  |  | ✓ |
| Statistical test for bimodal methylation | ✓ |  | ✓ |
| Fragment-level classification | ✓ |  |  |
| M-bias plot | ✓ | ✓ |  |
| CpH methylation calling |  | ✓ | ✓ |
| Collapse multiple samples and regions into a tabular format | ✓ |  | ✓ |
| Hydroxymethylation (5hmc) calling |  |  | ✓ |
| Oxford Nanopore Technology support | ✓ |  |  |
